# Ultrastructure and Enzymatic Hydrolysis of Deuterated Switchgrass

**DOI:** 10.1038/s41598-018-31269-w

**Published:** 2018-09-05

**Authors:** Samarthya Bhagia, Xianzhi Meng, Barbara R. Evans, John R. Dunlap, Garima Bali, Jihua Chen, Kimberly Shawn Reeves, Hoi Chun Ho, Brian H. Davison, Yunqiao Pu, Arthur J. Ragauskas

**Affiliations:** 1 Department of Chemical and Biomolecular Engineering, University of Tennessee, Knoxville, TN 37996 USA; 2 Advanced Microscopy and Imaging Center, University of Tennessee, Knoxville, TN 37996 USA; 3 Chemical Sciences Division, Oak Ridge National Laboratory**, Oak Ridge, TN 37831 USA; 4 Renewable Bioproducts Institute, School of Chemistry and Biochemistry, Georgia Institute of Technology, Atlanta, GA 30332 USA; 5 Center for Nanophase Materials Sciences, Oak Ridge National Laboratory, Oak Ridge, TN 37831 USA; 6 Carbon and Composite Group, Materials Science and Technology Division, Oak Ridge National Laboratory, Oak Ridge, TN 37831 USA; 7 The Bredesen Center for Interdisciplinary Research and Graduate Education, The University of Tennessee, Knoxville, TN 37996 USA; 8 Biosciences Division, Oak Ridge National Laboratory, Oak Ridge, TN 37831 USA; 9 Joint Institute of Biological Sciences, Biosciences Division, Oak Ridge National Laboratory, Oak Ridge, TN 37831 USA; 10 Center for Renewable Carbon, Department of Forestry, Wildlife, and Fisheries, University of Tennessee Institute of Agriculture, Knoxville, TN 37996 USA

## Abstract

Neutron scattering of deuterated plants can provide fundamental insight into the structure of lignocellulosics in plant cell walls and its deconstruction by pretreatment and enzymes. Such plants need to be characterized for any alterations to lignocellulosic structure caused by growth in deuterated media. Here we show that glucose yields from enzymatic hydrolysis at lower enzyme loading were 35% and 30% for untreated deuterated and protiated switchgrass, respectively. Lignin content was 4% higher in deuterated switchgrass but there were no significant lignin structural differences. Transmission electron microscopy showed differences in lignin distribution and packing of fibers in the cell walls that apparently increased surface area of cellulose in deuterated switchgrass, increasing cellulose accessibility and lowering its recalcitrance. These differences in lignification were likely caused by abiotic stress due to growth in deuterated media.

## Introduction

Structural components of plants, mainly cellulose, hemicellulose, and lignin, are enormous carbon resources that can be utilized for production of renewable fuels, chemicals, and materials. The complex structure of lignocellulosics that make up plant cell walls needs to be clearly understood for the success of these biorefining technologies^1^. Neutron scattering allows the study of structural and dynamic properties of lignocellulosic biomass at multiple length scales in a non-destructive manner^2^. Due to differences in scattering length density of hydrogen and deuterium, isotopic substitution of hydrogen with deuterium permits analysis of individual components in a system such as dynamic visualization of protein-carbohydrate and lignin-cellulose interactions^3^. Hydroponic cultivation of plants in deuterated media can achieve relatively high levels of deuterium substitution that is highly desired for neutron scattering studies due to the lessening of background scattering^4,5^. Deuterium substitution in switchgrass (*Panicum virgatum*) was achieved by growing it first in H_2_O for one to three months followed by transferring hydroponic plants grown from tiller cuttings to a 1:1 solution of D_2_O:H_2_O containing Schenk and Hildebrandt’s basal salts^4^. Approximately 34% deuterium incorporation was calculated from ^2^H/^1^H ratio from ^2^H and ^1^H NMR spectra of deuterated switchgrass grown in 50% D_2_O medium^4,6^. Switchgrass cultivated by this method exhibited gross morphology and growth rates similar to controls grown in H_2_O^4^, in contrast to the slower growth and dwarfing observed for winter rye^7^, annual rye grass^8^, Arabidopsis^9,10^, and other species^11^. Such deuterated plants for fundamental studies need to be investigated for any possible alterations to physical and chemical characteristics due to stress caused by growth in deuterated media.

In this study, deuterated switchgrass with 34% isotopic substitution (grown in 50% D_2_O medium) and protiated switchgrass (H_2_O control) were investigated for glucose yields by enzymatic hydrolysis by *T. reesei* cellulases. It is known that higher binding energy and shorter bond length of deuterium bonds compared to hydrogen bonds result in slower reaction rates, a phenomenon known as the kinetic isotope effect (KIE)^3^. However, deuterated switchgrass had higher glucose yield from enzymatic hydrolysis than protiated switchgrass. Thus, this study characterized these plants to find the cause of higher glucose yields from enzymatic hydrolysis of deuterated switchgrass and better understand cell wall recalcitrance when plants are subjected to environmental stress.

## Findings

**Figure 1:**
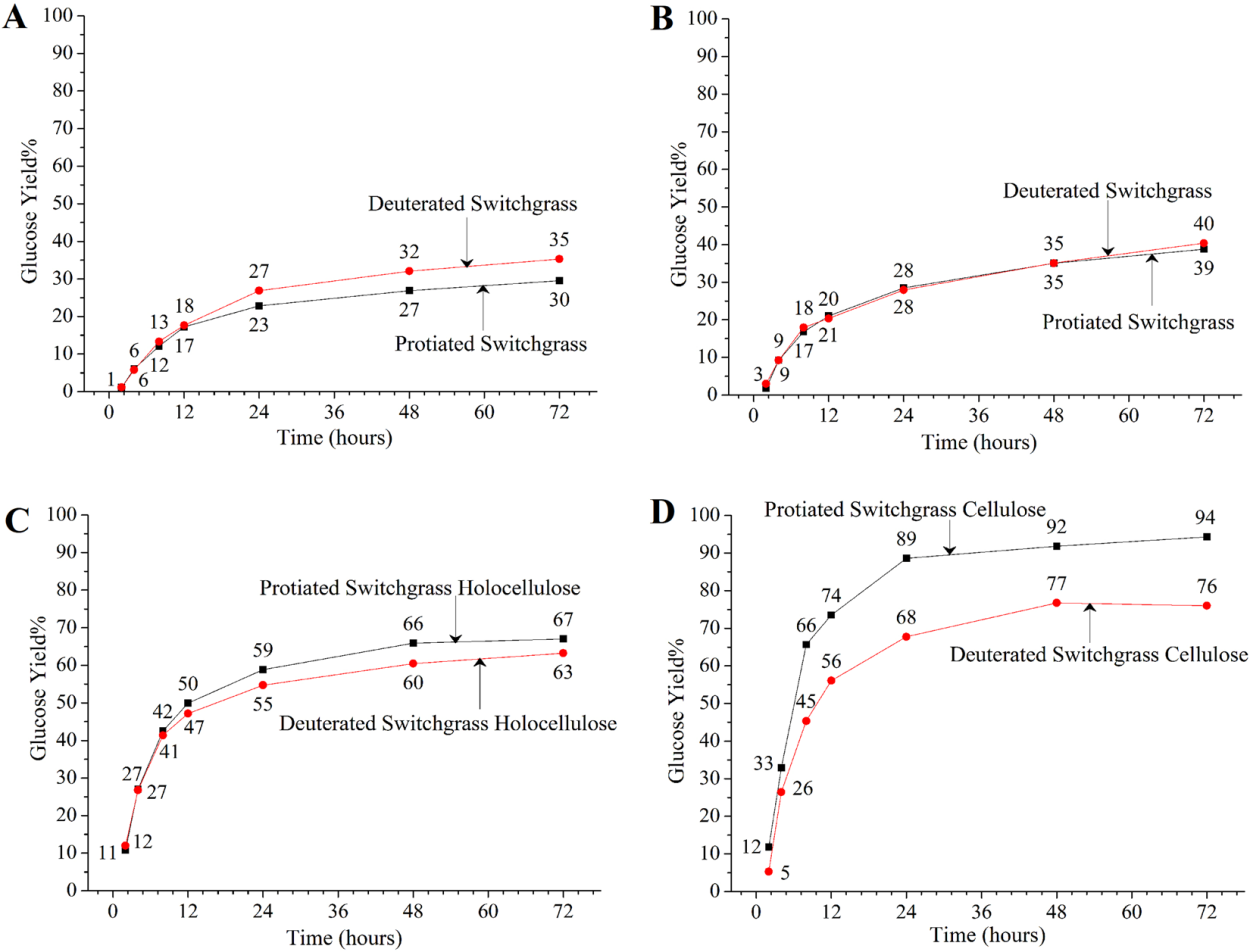
Glucose yields from enzymatic hydrolysis. Graphs show glucose yields from enzymatic hydrolysis of switchgrass at (**A**) 20 FPU cellulase +40 CBU β-glucosidase and (**B**) 40 FPU cellulase +80 CBU β-glucosidase per gram glucan, and (**C**) holocellulose and (**D**) cellulose isolated from switchgrass at an enzyme loading of 20 FPU cellulase +40 CBU β-glucosidase per gram glucan, in their protiated (square) and deuterated (circle) forms, respectively, over 72 h of reaction.

KIE was expected to lower enzymatic hydrolysis yields of deuterated switchgrass compared to protiated switchgrass. However, enzymatic hydrolysis of deuterated switchgrass resulted in about 5% higher glucose yield at a loading of 20 FPU cellulase +40 CBU *β*-glucosidase per g glucan in switchgrass than protiated switchgrass after 72 h of reaction (Fig. 1). At a higher enzyme loading of 40 FPU cellulase +80 CBU *β*-glucosidase per g glucan in switchgrass, glucose yields were similar (40%) between these two types of switchgrass. To understand why deuterated switchgrass did not show lower yield in enzymatic hydrolysis, switchgrass samples were delignified to recover protiated and deuterated holocellulose (cellulose + hemicellulose). This was followed by protiated and deuterated α-cellulose recovery through alkaline removal of hemicellulose from holocellulose samples. Deuterated holocellulose and α-cellulose isolated from switchgrass had lower enzymatic hydrolysis glucose yields after 72 h of reaction than their protiated counterparts by about 4 and 18 percentage points, respectively, at a loading of 20 FPU (filter paper units) cellulase +40 CBU (cellobiose units) *β*-glucosidase per gram glucan in switchgrass (Fig. 1). Thus, enzymatic hydrolysis of deuterated holocellulose and cellulose showed the expected kinetic isotope effect. These data indicated that lignin is likely responsible for better or similar glucose yield from deuterated switchgrass than protiated switchgrass through enzymatic hydrolysis. As it took several months to grow these samples by cultivation of a limited number of switchgrass plants in hydroponic chambers, the amount of material available for enzymatic hydrolysis was limited. Additionally, since the tillers were harvested after one to two months growth, moisture content of the never-dried biomass was relatively high, about 80%, as compared to field-grown end-of-season harvested switchgrass. Therefore, only enzymatic hydrolysis of switchgrass was repeated at the high enzyme loading to confirm the unexpected outcome of similar or higher yield by deuterated switchgrass, as the other samples (holocellulose and α-cellulose) showed the expected kinetic isotope effect. At 40 FPU + 80 CBU enzyme loading, the glucose yields from protiated switchgrass from two biological replicates were 38.76% and 38.80% while that from deuterated switchgrass were 40.35% and 39.01%, confirming the accuracy of the results.

**Figure 2:**
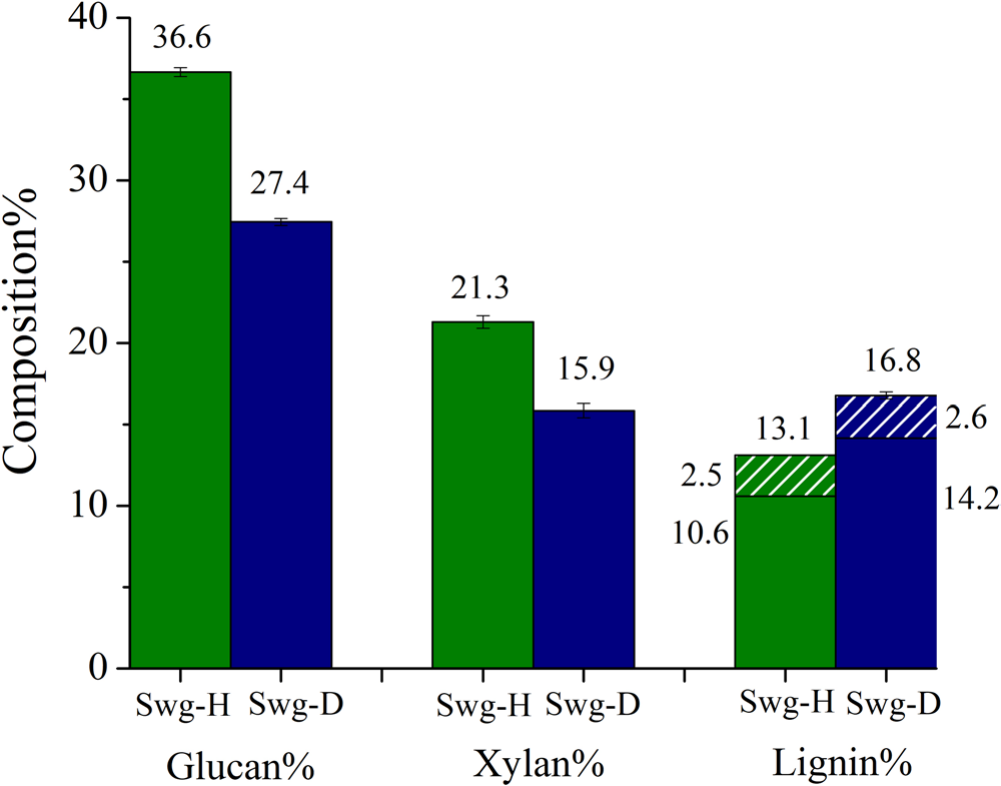
Glucan, xylan and lignin composition of protiated and deuterated switchgrass. Lignin content is sum of acid insoluble lignin (solid) and acid soluble lignin (pattern).

Compositional analysis of switchgrass showed that lignin content was higher for deuterated switchgrass (16.8%) than protiated switchgrass (13.1%) (Fig. 2). This is contrary to the higher glucose yield by deuterated switchgrass as elevated lignin content can negatively affect deconstruction of plant polysaccharides by cellulase enzymes^12,13^. For example, poplar wood released three times more sugars with enzymatic hydrolysis due to reduced lignin content of the tension wood region^14,15^. Moreover, glucan and xylan content of deuterated switchgrass were about 9 and 5 percentage points lower than protiated switchgrass. In our recent article, characterization of lignin isolated after enzymatic digestion of these two switchgrass samples was carried out^16^. Molecular weight analysis showed that deuterated lignin had slightly higher weight-average molecular weights (11109 g/mol) than protiated lignin (9009 g/mol), likely due to the higher atomic weight of deuterium. Considering lignin monomer mol. wt. as 203 g/mol based on S/G ratio of 0.77^16^ found in the earlier study, degree of polymerization increased roughly from 44 to 54. If equal isotopic substitution in all biomass components is assumed, i.e., 34% deuterium incorporation in lignin, and 10–13 hydrogen atoms per monomer, the expected increase in the mol. wt. of lignin will only be about 82 g/mol in switchgrass grown in 50% D_2_O. Moreover, C-D and O-D bond lengths are slightly smaller than C-H and O-H^17,18^, suggesting that size exclusion chromatography may show slightly lower or similar molecular weight of deuterated switchgrass lignin compared to its protiated counterpart. This issue will be investigated in the future by using absolute detection instead of calibration using polystyrene standards to see whether this increase was actually caused by plant biosynthesis.

Two-dimensional heteronuclear single quantum coherence (HSQC) NMR of the deuterated and protiated lignin and ^31^P NMR analysis of hydroxyl groups in lignin suggested that there were no distinct effects of deuteration on core structure of lignin. Phenolic groups of lignin can affect its hydrophobicity which is an important factor in unproductive binding of enzyme to lignin that leads to less active enzyme available for cellulose saccharification^19,20^. Similar phenolic hydroxyl content among the two isotopic samples indicated no significant difference in their hydrophobicity that might have affected yields from enzymatic hydrolysis.

**Figure 3:**
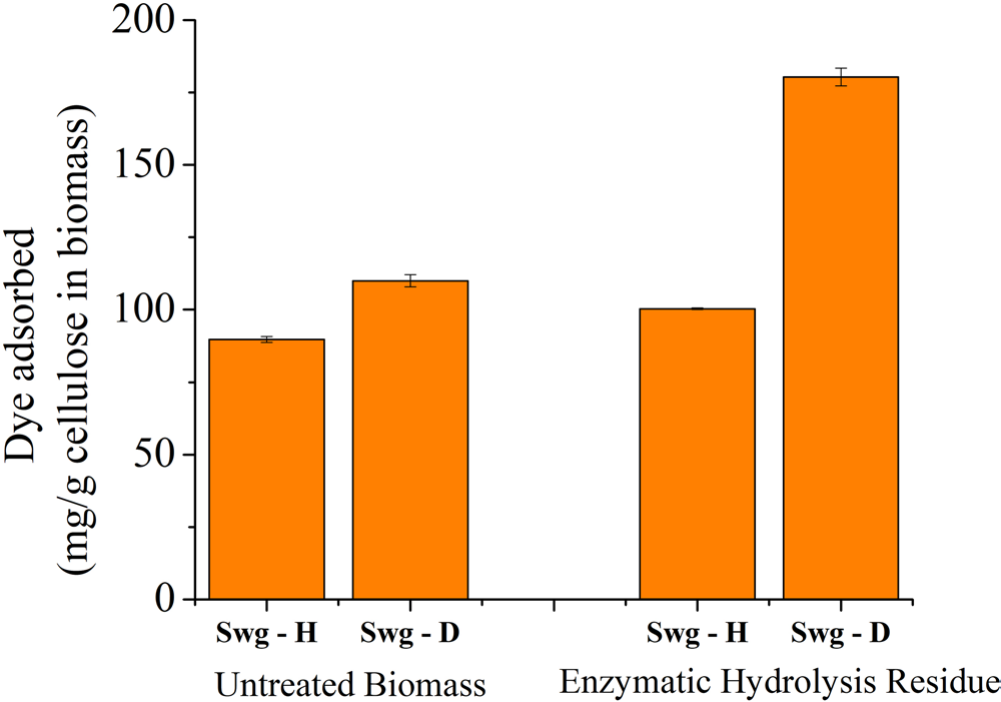
Dye adsorption onto cellulose in untreated and enzymatic hydrolysis residues of protiated and deuterated switchgrass.

**Figure 4:**
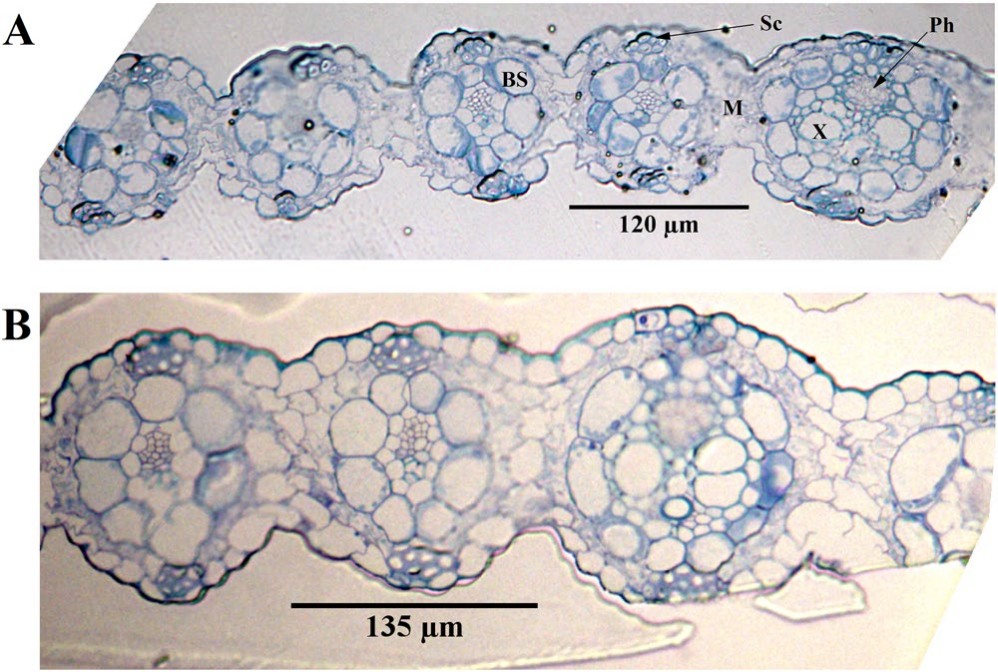
Bright-field images of transverse section of protiated (**A**) and deuterated (**B**) switchgrass stained with toluidine blue at magnification of 25X. Sc: Sclerenchyma, BS: Bundle Sheath, M: Mesophyll, X: Xylem and Ph: Phloem.

**Figure 5:**
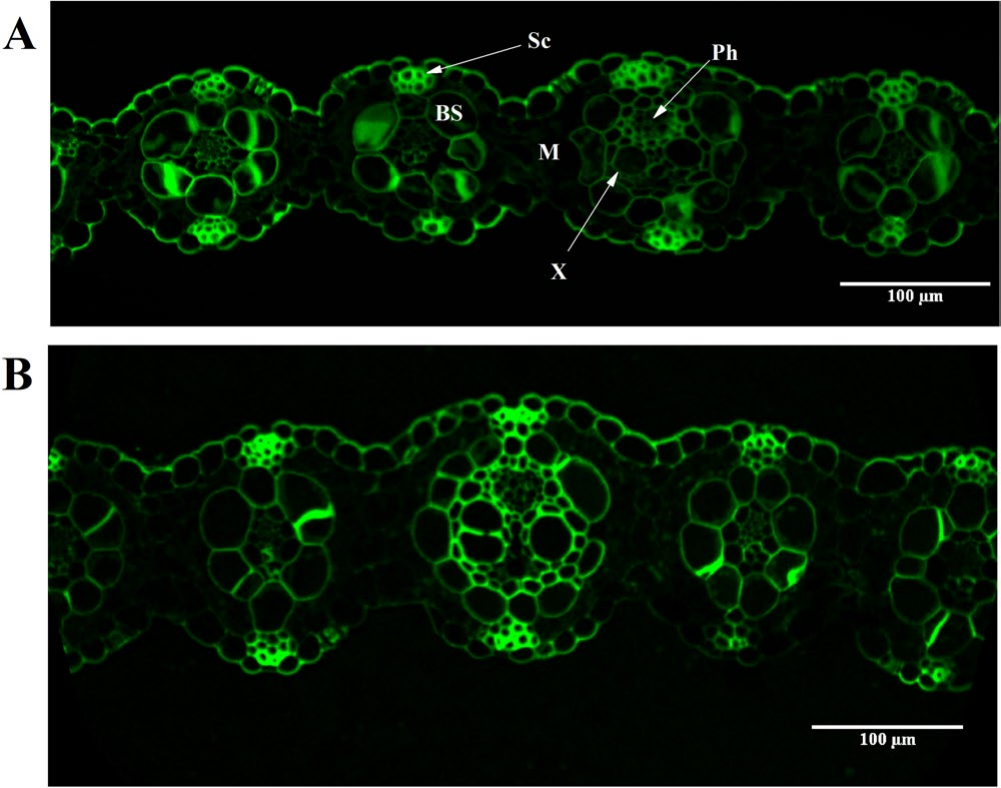
Confocal microscopy images showing autofluorescence of lignin of transverse section of protiated (**A**) and deuterated switchgrass (**B**) at magnification of 25X. Sc: Sclerenchyma, BS: Bundle Sheath, M: Mesophyll, X: Xylem and Ph: Phloem.

**Figure 6:**
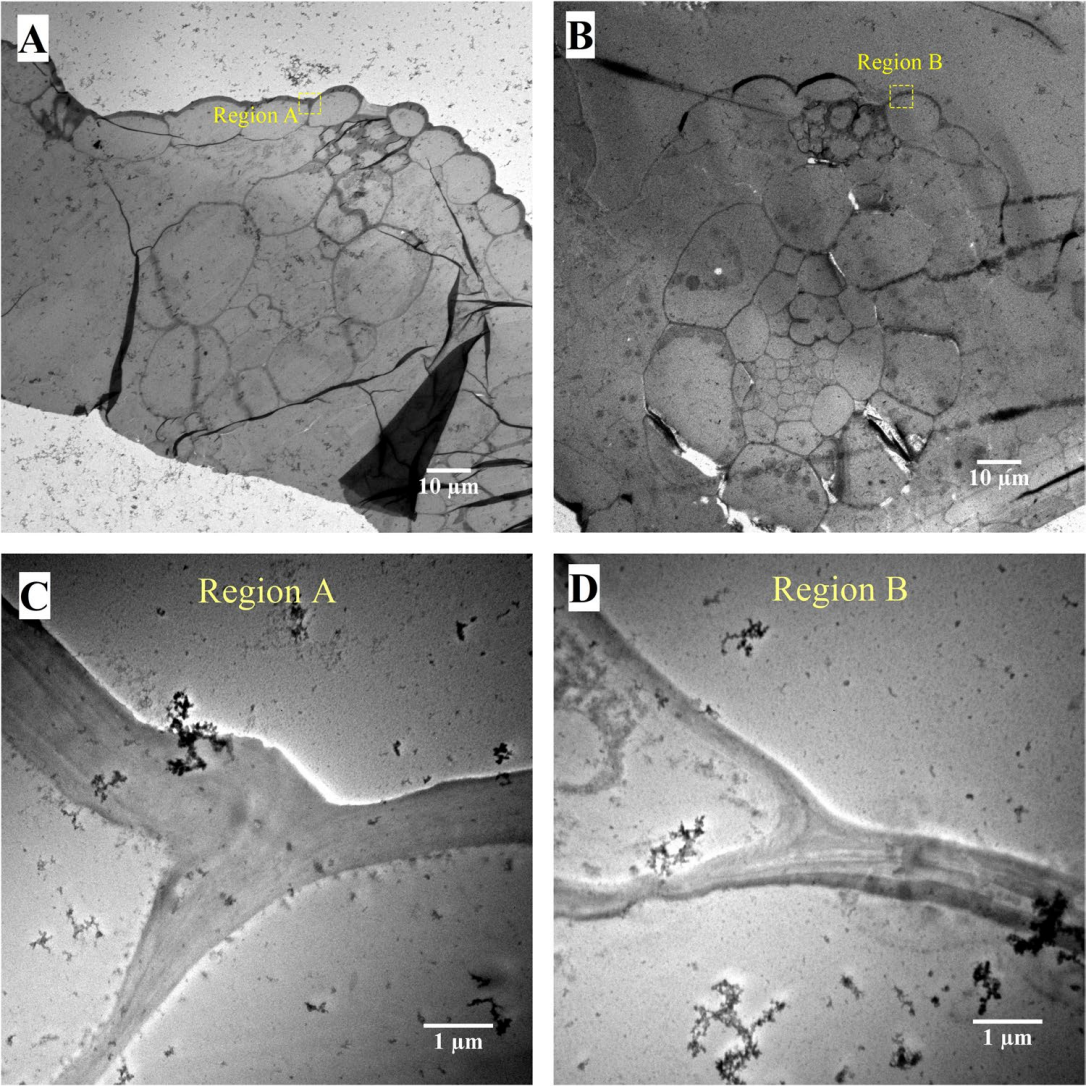
TEM images of transverse sections of protiated (**A**,**C**) and deuterated switchgrass (**B**,**D**). Images (**C**,**D)** show magnified regions for the highlighted regions (**A** and **B**) in images (**A** and **B**) respectively. Scale bars: (**A**,**B**) – 10 µm and (**C**,**D**) – 1 µm.

**Figure 7:**
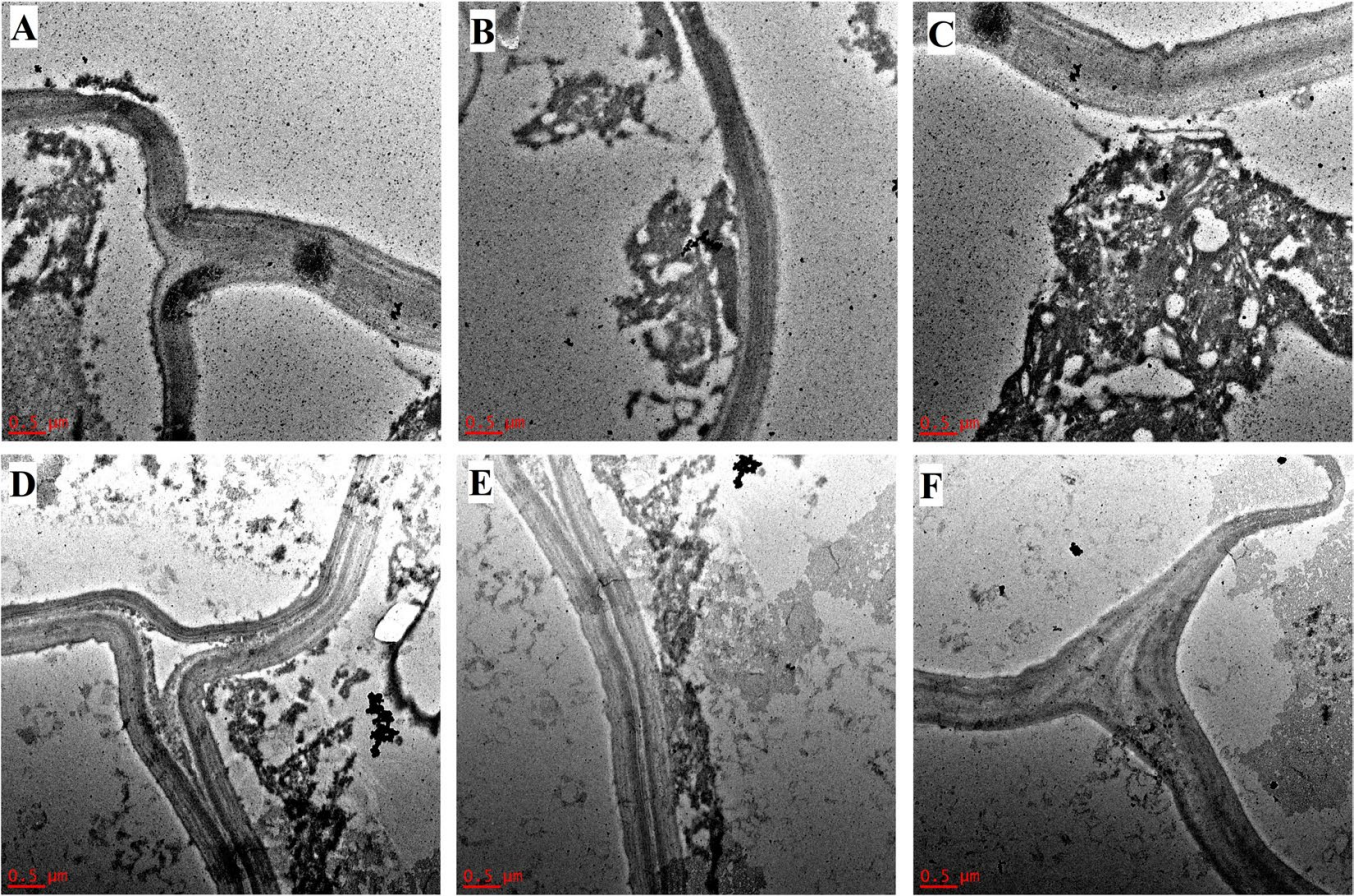
TEM images of transverse sections of protiated (**A**–**C**) and deuterated switchgrass (**D**–**F**) stained with KMnO_4_. Scale bar: 0.5 µm.

Modified Simons’ staining technique was then used to estimate cellulose accessibility in switchgrass. Cellulosic dye Direct Orange 15 was ultra-filtrated to recover a high molecular (HW) weight fraction (>100 KDa). Cellulose accessibility measured from adsorption of this high molecular weight orange dye fraction has been correlated to enzymatic hydrolysis yields, due to similar size of the enzyme and HW of dye fraction^21,22^. Results showed that dye adsorption based on per g glucan in biomass was significantly higher for deuterated switchgrass than that in protiated switchgrass both before, and after enzymatic hydrolysis at high enzyme loading (Fig. 3). Thus, higher cellulose accessibility was the primary reason for the inverse isotope effect in enzymatic hydrolysis of switchgrass. Further, bright-field, fluorescence and electron microscopy were carried out to see how lignin may have affected cellulose accessibility. Thin switchgrass transverse sections were stained with Toluidine blue and observed under a light microscope. Toluidine blue stains lignified cell walls blue-green and non-lignified cell walls reddish purple^23^. Lignification was further studied on a confocal microscope using the auto-fluorescence property of lignin^24^. Figures 4 and 5 show a typical Kranz anatomy of bundle sheath arrangement and mesophyll cells for C_4_ photosynthesis in switchgrass^25^. Deuterated switchgrass had a normal morphology. Bundle sheath, sclerenchyma and xylem cell walls from both types of switchgrass were stained blue and fluoresced under UV light. Both samples showed low lignification of mesophyll and phloem cell walls. Further, distribution of lignin in deuterated and protiated switchgrass cell walls was studied by transmission electron microscopy (TEM) by staining with potassium permanganate (KMnO_4_) that has a high affinity to lignin compared to polysaccharides in plant cell walls. It oxidizes phenolic groups in lignin and precipitates as manganese dioxide at the reaction site^26^. This electron-dense stain for TEM was applied as a post-stain on thin sections. Darker regions in TEM images represent higher concentration of MnO_2_ and thus, higher presence of lignin in plant cell walls. Figures 6 and 7 show that cell walls of deuterated switchgrass had lignin condensed in specific regions forming light and dark patterns compared to the higher uniformity in lignin distribution in protiated switchgrass cell walls. The images of deuterated switchgrass also show somewhat lower packing of lignocellulosic fibers that may have contributed to increase in accessibility. Moreover, KMnO_4_ strongly stained cytoplasmic material of plant cells. This is the first example of alterations in cell wall lignification of deuterated plants. Nevertheless, some changes to ultrastructure of plants from D_2_O growth have been reported before. Previous studies that carried out imaging of rye seedlings and duckweed grown in D_2_O focused on internal cellular structures. TEM images of winter rye seedlings germinated in H_2_O for 2 days and 99.8% D_2_O^7^ for 9 days found differences in chloroplast structure. Growth in 50% or 63% D_2_O caused disorganization of cellular structure of fronds of *Lemna perpusilla* duckweed^27^ TEM images showed disruption of the ultrastructure of the tonoplast and chloroplast membranes of *Lemna minor* during initial exposure to 50% D_2_O followed by recovery and adaptation after 24 hours^7,27,28^.

Localization of lignin in specific regions of cell walls left some regions with reduced lignification while others with increased concentration of lignin even when the lignin content of bulk material was elevated. Lower packing of fibers and regions of reduced lignification contributed to higher dye adsorption and hence, higher enzyme accessibility to cellulose that overpowered the kinetic isotope effect. Hemicellulose and lignin in plant cell walls prevent cellulase enzymes from efficiently converting cellulose to cellobiose (*β*-glucosidase converts cellobiose to glucose) due to low number of substrate binding sites and extensive linkages between these polymers. Moreover, cellulases are inactivated over time of reaction^29^. At the higher enzyme loading, the excessive amount of active cellulases can remove several glucan chains and create more cellulose substrate binding sites, as evidenced by higher accessibility of the enzymatic hydrolysis residues. However, a plateau is reached in this process as hemicellulose and lignin prevent further breakdown of cellulose. At the lower enzyme loading, while KIE reduced enzymatic conversion, the higher number of accessible substrate sites resulted in higher glucose yields from deuterated switchgrass. However, even at higher enzyme loading, hemicellulose and lignin prevented cellulose conversion beyond ~40%. More cellulose surfaces were rendered accessible in protiated switchgrass over reaction time at higher enzyme loading than at lower enzyme loading, resulting in elevation of its cellulose conversion to a level similar to deuterated switchgrass. Irregular or abnormal lignification has been previously found in plants under environmental stress. It has been suggested that abiotic stresses increase lignification of cell walls^30^. Stress due to growth in deuterated media likely led to elevation of lignin content and abnormal distribution of lignin. Yang *et al*.^31^ found that the genes that had increased expression in Arabidopsis due to growth in D_2_O were stress related and included oxidative stress, wound, defense, water deprivation and temperature shock genes. Donaldson reported irregular lignification and abnormality in cell walls of drought affected pine trees (*Pinus radiata*). In these trees, severely reduced lignification was found in collapsed cell walls^32^. Oxidative stress affects peroxidase activity that can increase lignification of cell walls^33^. However, physiological and metabolic changes from growth in high D_2_O concentrations are still not completely understood and we continue to study these phenomena.

These results illustrate that lignification patterns and packing fibers in plant cell walls can reduce recalcitrance of biomass despite higher lignin content. Although much emphasis has been placed on the relationship between the structure of cellulose, hemicellulose and lignin in terms of recalcitrance, these results clearly demonstrate that even when these components are comparable, changes in recalcitrance can still occur due to the ultrastructure of the plant cell wall. In the future, these results suggest that we may be able to engineer plants with reduced recalcitrance by altering the location of lignin within the plant cell wall structure while maintaining the same bulk distribution of lignin, cellulose and hemicellulose. Given the interest and future needs for low recalcitrance biomass these results provide a new insight into engineering energy crops.

## Methods

### Hydroponic Cultivation of Switchgrass

Switchgrass was grown hydroponically from tiller cuttings using an in-house assembled perfusion system as described previously^4,5^. Switchgrass seeds (*Panicum virgatum*, Alamo cultivar) were obtained from the Bioenergy Science Center located at Oak Ridge National Laboratory, Oak Ridge, Tennessee, U. S. A. Deuterium oxide (D_2_O), 99.8%, was purchased from Cambridge Isotope Laboratories (Cambridge, Massachusetts). Distilled water was further purified by filtration through a Barnstead E-Pure water purification system before use. The growth media were prepared with Schenk and Hildebrandt’s basal salts (without sugars, vitamins, or rooting hormones). Plant jars, filter-vented lids, and Schenk and Hildebrandt’s basal salt mixture were purchased from Phytotechnology Laboratories (Shawnee Mission, Kansas, U. S. A.). Switchgrass was grown from seed in commercial potting soil. Tillers were cut from the root crowns of the established switchgrass and incubated in plant jars containing sterile Schenk and Hildebrandt’s basal salts in H_2_O until appearance of roots after 5–10 days. The rooted tiller cuttings were transferred to growth chambers assembled from 1 L graduated cylinders fitted with rubber closures with inflow and outflow tubes perfused with dry ambient air using an aquarium pump and in-line desiccant tubes as described previously^8^. After one to eleven months growth, hydroponic plants were transferred to growth medium containing 50% D_2_O for deuteration, while control plants were maintained in H_2_O medium. Samples for microscopy were taken from 30–50 cm tall tillers harvested after one to two months growth from the hydroponic plants, with deuterated plants harvested 1 to 6 months after transfer to 50% D_2_O.

### Composition of switchgrass

Switchgrass samples were dried overnight in a fume hood. This was followed by size reduction in a Mini Wiley Mill (Thomas Scientific, Swedesboro, NJ) through a screen size of ASTM standard mesh no. 40. Samples were extracted with toluene:ethanol (2:1 v/v) in a soxhlet apparatus for 8 h. Composition of glucan, xylan, and lignin in protiated and deuterated switchgrass were determined by standard National Renewable Energy Laboratory (NREL) procedure “Determination of Structural Carbohydrates and Lignin in Biomass”^34^. Acid soluble lignin was determined by measuring absorbance at 320 nm in a UV-vis spectrophotometer (Perkin Elmer, Lambda 35) using extinction coefficient of 30 L/(g.cm).

### Holocellulose Recovery from Switchgrass

Extractive-free biomass samples (~0.6 g) were mixed with peracetic acid (~2.1 g) and DI water (~5.8 mL)^35^. This mixture was then stirred at 25 °C for 24 h in dark followed by repeated centrifugation and washing with DI water to isolate the holocellulose samples.

### Cellulose Recovery from Switchgrass

Cellulose was isolated from holocellulose samples. Holocellulose samples (~0.10 g) were suspended in 17.5 wt% NaOH solution (~5.00 mL) at 25 °C for 2 h. The mixture was then diluted to 8.75% NaOH solution by adding ~5.00 mL of DI water and allowed to stir for additional 2 h. The isolated α-cellulose samples were then recovered from centrifugation, washed with 50 mL of 1% acetic acid and an excess of DI water until the pH of the filtrate was close to 7.

### Enzymatic Hydrolysis

Enzymatic hydrolysis procedure was adapted from NREL LAP “Enzymatic Saccharification of Lignocellulosic Biomass”^36^. Glucan loadings of solids was 0.1%. The samples were never dried before enzymatic hydrolysis to avoid pore collapse. Enzymatic hydrolyses were performed in Erlenmeyer flasks in 50 mM citrate buffer (pH 4.8) and 1% (v/v) of antibiotic antimycotic solution (Sigma Aldrich cat# A5955). Enzymes used were cellulase from *Trichoderma reesei* ATCC 26921 (Sigma-Aldrich Corp. in St. Louis, MO) and *β*-glucosidase from almonds (Sigma-Aldrich, cat# G0395). Enzyme loading for cellulose and holocellulose recovered from switchgrass, and switchgrass at lower enzyme loading was 20 FPU (filter paper units) cellulase +40 CBU (cellobiose units) *β*-glucosidase per gram glucan. High enzyme loading for switchgrass was 40 FPU cellulase +80 CBU *β*-glucosidase per gram glucan. The mixture was incubated at 50 °C with shaking at 150 rpm for 72 hours. The reaction was stopped by quenching the aliquots for 10 min in a boiling water bath followed by centrifugation (MiniSpin Plus, Eppendorf AG, Hauppauge, NY) at 10,000 rpm for 5 minutes. The liquid supernatants were then frozen to −20 °C until sugar quantification. Supernatants were diluted, filtered and injected into high-performance anion exchange chromatography with pulsed amperometric detection (HPAEC-PAD) using Dionex ICS-3000 (Dionex Corp. in Sunnyvale, CA) equipped with an electrochemical detector, a guard CarboPac PA1 column (2 × 50 mm, Dionex), a CarboPac PA1 column (2 × 250 mm, Dionex), a AS40 automated sampler and a PC 10 pneumatic controller at room temperature. 0.20 M and 0.40 M NaOH was used as the eluent and post-column rinsing effluent. The total analysis time was 70 min, with a flow rate 0.40 mL/min. Calibration was performed with external standard solutions of glucose and xylose, and fucose as an internal standard.

### Modified Simons’ Staining Technique

The modified Simons’ staining technique was performed according to Chandra *et al*.^21^. 1% Direct Orange 15 dye (Sigma Aldrich, St. Louis, MO) solution in water was ultrafiltered at 4000 rpm for 30 min through 100 KDa membrane (Amicon Ultra-15 UFC910024, EMD Millipore, Burlington, MA). The high molecular weight fraction was used for analysis. 1% solids on dry basis, 0.1% dye, 1% NaCl and 50 mM potassium phosphate buffer solution were shaken in serum vials at 70 °C for 24 hours. The samples were centrifuged, supernatants diluted and absorption was measured at 445 nm by a UV-Vis spectrophotometer (Perkin Elmer Lambda 20, Akron, OH).

### Microscopy

Small leaf cuttings were fixed with 2.5% glutaraldehyde (Electron Microscopy Sciences, Hatfield, PA) and 2% formaldehyde solution (Fisher Scientific, Waltham, MA) in 0.1 M sodium cacodylate buffer (Electron Microscopy Sciences, Hatfield, PA) for 1 h at RT under slight vacuum. The pieces were then removed from fixative and washed three times in 0.1 M cacodylate buffer. This solution was replaced consecutively by 10, 30, 50, 70, 90, 100, and 100% v/v ethanol in water, each for 10 minutes. This was followed by infiltration with 25, 50, 75, 100, and 100% v/v medium-grade LR White (Electron Microscopy Sciences, Hatfield, PA) solution in ethanol each for 8 hours at RT. Each resin infiltrated sample was then placed in a gelatin capsule and filled completely with pure LR White. Curing was done at 60 °C for 24 h in a gravity convection oven^37^. Transverse sections of resin blocks were taken on Leica UM7 ultramicrotome (Leica Microsystems Inc. in Buffalo Grove, IL) in slices of approximately 500 nm for light and confocal microscopy and 100 nm for TEM. Brightfield microscopy was carried out on Nikon Eclipse E600 on sections stained with 0.02% Toluidine Blue for 10 minutes. Confocal microscopy was carried out on Leica SP8 with excitation using 405 nm laser and detection at 510–570 nm for auto-fluorescence of lignin. For TEM, slices were transferred to 100 mesh copper grids, followed by staining with 1% potassium permanganate (KMnO_4_) in distilled water for 10 minutes and washed with distilled water. The stained samples were examined in a Zeiss Libra 120 TEM (Carl Zeiss Microscopy, Thornwood, NY) equipped with in-line Omega filter at 120 kV. A LaB6 filament with an emission current of ~6 μA was used to ensure minimal morphological changes of the samples during TEM imaging. ImageJ software^38^ was used to adjust brightness, contrast, addition of scale bars and labeling.

## Data Availability

All data generated or analyzed during this study are included in this published article.
